# Shaping the Dynamics of a Bidirectional Neural Interface

**DOI:** 10.1371/journal.pcbi.1002578

**Published:** 2012-07-19

**Authors:** Alessandro Vato, Marianna Semprini, Emma Maggiolini, Francois D. Szymanski, Luciano Fadiga, Stefano Panzeri, Ferdinando A. Mussa-Ivaldi

**Affiliations:** 1 Department of Robotics, Brain and Cognitive Sciences, Istituto Italiano di Tecnologia, Genova, Italy; 2 Department of Human Physiology, University of Ferrara, Ferrara, Italy; 3 Center for Neuroscience and Cognitive Systems, Istituto Italiano di Tecnologia, Rovereto, Italy; 4 Institute of Neuroscience and Psychology, University of Glasgow, Glasgow, United Kingdom; 5 Department of Physiology, Northwestern University, Chicago, Illinois, United States of America; 6 Department of Biomedical Engineering, Northwestern University, Evanston, Illinois, United States of America; 7 Sensory Motor Performance Program, Rehabilitation Institute of Chicago, Chicago, Illinois, United States of America; University College London, United Kingdom

## Abstract

Progress in decoding neural signals has enabled the development of interfaces that translate cortical brain activities into commands for operating robotic arms and other devices. The electrical stimulation of sensory areas provides a means to create artificial sensory information about the state of a device. Taken together, neural activity recording and microstimulation techniques allow us to embed a portion of the central nervous system within a closed-loop system, whose behavior emerges from the combined dynamical properties of its neural and artificial components. In this study we asked if it is possible to concurrently regulate this bidirectional brain-machine interaction so as to shape a desired dynamical behavior of the combined system. To this end, we followed a well-known biological pathway. In vertebrates, the communications between brain and limb mechanics are mediated by the spinal cord, which combines brain instructions with sensory information and organizes coordinated patterns of muscle forces driving the limbs along dynamically stable trajectories. We report the creation and testing of the first neural interface that emulates this sensory-motor interaction. The interface organizes a bidirectional communication between sensory and motor areas of the brain of anaesthetized rats and an external dynamical object with programmable properties. The system includes (a) a motor interface decoding signals from a motor cortical area, and (b) a sensory interface encoding the state of the external object into electrical stimuli to a somatosensory area. The interactions between brain activities and the state of the external object generate a family of trajectories converging upon a selected equilibrium point from arbitrary starting locations. Thus, the bidirectional interface establishes the possibility to specify not only a particular movement trajectory but an entire family of motions, which includes the prescribed reactions to unexpected perturbations.

## Author Summary

Brain-machine interfaces establish new communication channels between the brain and the external world with the goal of restoring sensory and motor functions for people with severe paralysis or sensory impairments. Current methodologies are based on decoding the motor intent from the recorded neural activity and transforming the extracted information into motor commands to control external devices as robotic arms. We developed a novel computational approach, based on the concept of programming dynamical behaviors trough the bi-directional sensory-motor interaction between the brain and the connected external device. This approach is based on the emulation of some control features of a biological interface, the spinal cord. The first prototype of our interface controls the state of motion of a simulated point mass in a viscous medium. The position of the point mass is encoded into a stimulus to the somatosensory cortex of an anesthetized rat. The evoked activity of a population of motor cortical neurons is decoded into a force vector applied to the point mass. The parameters of the encoder and of the decoder are set to approximate a desired force field. In the first test of the interface, we obtained a family of trajectories that converged upon a stable attractor.

## Introduction

In a recent demonstration [1], Schwartz and coworkers decoded neural activities from the motor area of a monkey's cerebral cortex to control the movement of a robotic arm. The monkey learned to activate the recorded neurons and to guide the arm for transporting food to the mouth. This is an undisputed milestone in Neural Engineering, highlighting the potential of neural interfaces (NIs) as a means to restore a connection with the world for people with severe paralysis. In addition to their clinical impact, NIs have the potential to revolutionize our ways to study the nervous system, by connecting live neural populations with external devices, both physical and simulated. This constitutes a leap forward with respect to current paradigms, in which physiological experiments and computational analyses are carried out separately.

Both the clinical and the basic science applications of NIs call for the possibility to close the sensory-motor loop, by combining a decoding interface – mapping neural activities into inputs to the external device – with an encoding interface – mapping the state of the device into a direct input to the brain, such as an electrical stimulus. In this study we addressed the challenge to create a coordinated bidirectional brain-machine interaction by concurrently setting up a decoding and an encoding interface, which combined generate a dynamic control policy in the form of a force field. In this approach, we aimed at emulating the operation of the spinal cord, as the prime biological interface between the brain and the musculoskeletal apparatus.

Ascending tracts of the spinal cord inform the brain about the state of motion of the limbs and about physical properties of the environment. Descending tracts distribute motor commands across groups of muscles both by direct connections with the motoneuronal pools and by connections with spinal interneurons that activate multiple muscles spanning one or more joints [2], [3]. Earlier studies in frogs [4]–[6], rats [7], and cats [8] have revealed that the electrical stimulation of the grey matter in the lumbar spinal cord results in a field of forces acting on the ipsilateral hind limb. This finding has a simple biomechanical basis. The force generated by a muscle varies depending on the state of motion of the muscle – i.e. its instantaneous length and shortening rate. In addition, variety of other factors, such as fatigue and hysteresis, and environmental variable, such as temperature, affect muscle force. While the detailed analysis of these factors is beyond the scope of this work, we may simply state that when the spinal cord activates an ensemble of muscles in response to a cortical command, the net mechanical outcome is a spatial pattern of forces – a force field – that sets the limb in motion. The above mentioned studies have highlighted the presence of convergent patterns of forces, but evidence from other investigations [9] have suggested more complex spatio-temporal structures of the underlying force fields.

Our study aimed at reproducing in an artificial interface this basic control mechanism. We considered the problem of generating by function approximation a force field that converges to a central equilibrium point. This is a very particular instantiation out of a much larger repertoire of possible mechanical behaviors, which may be represented as a functional map from the state of motion of a limb, i.e. its position and velocity, and the ensuing force generated by the musculoskeletal apparatus.

## Results

### General scheme of the dynamic Neural Interface

In the language of control theory, the spinal cord establishes a policy [10] by specifying the forces to be generated throughout the reachable space in response to unexpected perturbations. We have adopted this perspective for developing a new type of neural interface called dynamic Neural Interface (dNI), which borrows a local portion of cortical tissue to emulate the generation of force fields by the spinal cord [4].

**Figure 1:**
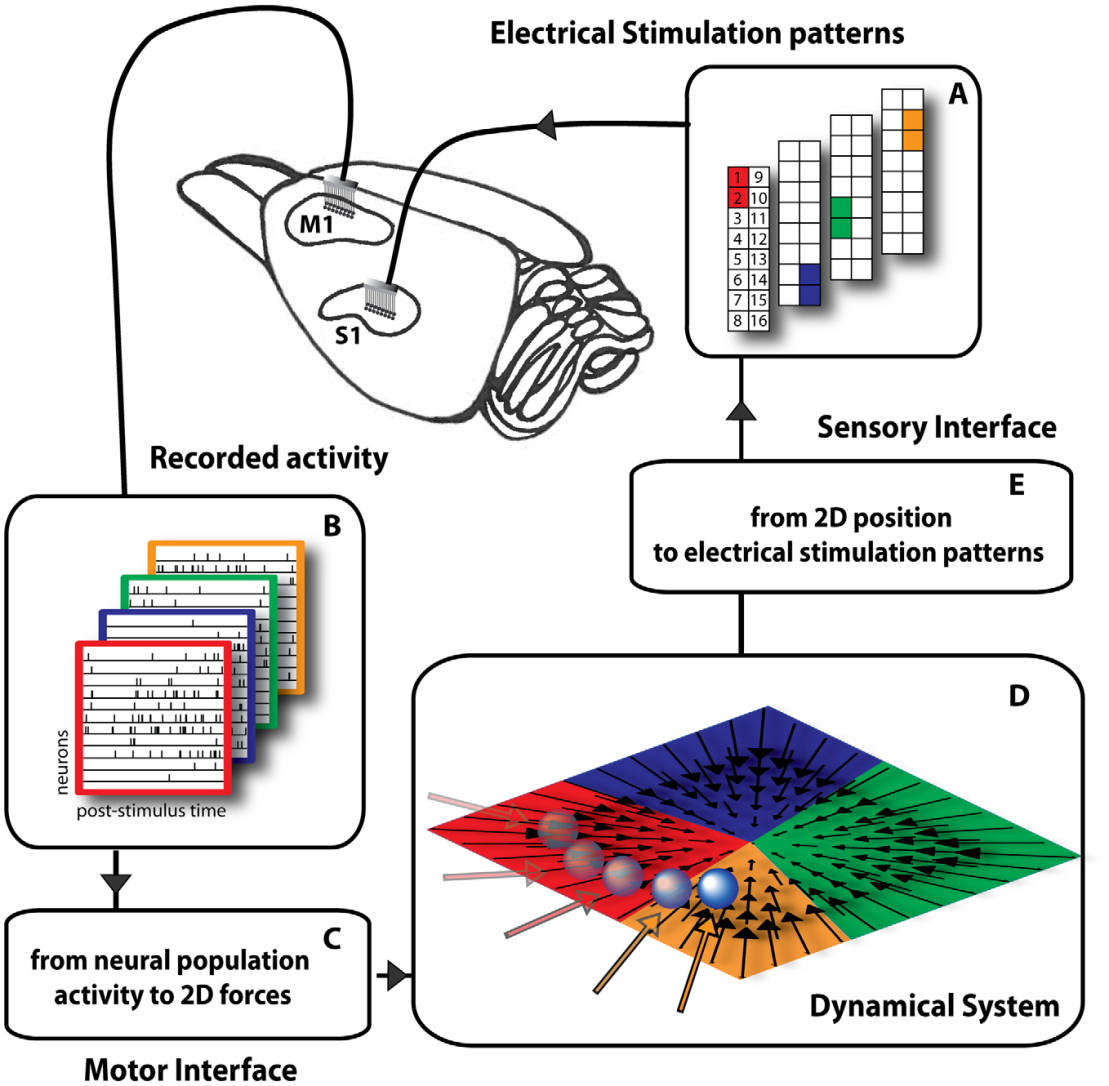
Experimental setup of the dynamic neural interface. We placed two 16-channel microwire arrays (*recording* and *stimulating* arrays) in the vibrissa motor (M1) and sensory areas (S1) of a rat brain cortex. (A) In this example 4 electrical stimulation patterns are set by specifying the pair of electrodes in the 16-channel microwire stimulating array placed in area S1. (B) The activity of a small population of single neurons (11 in this illustration) of area M1 is recorded in response to each electrical stimulation pattern. The activity of each neuron is plotted on a row over a rectangular frame, whose color indicates the correspondence with a stimulation pattern. (C) The motor interface generates a force vector from the first two principal components of the response of the M1 neurons. (D) The obtained force vector is applied to a simulated point-mass moving in a viscous medium. The interaction with such dynamical system aims to emulate a reaching movement creating a convergent force field similar to the force fields observed during microstimulation of the spinal gray matter. (E) The sensory interface maps each point in the field into the corresponding stimulation pattern.

The dNI has 4 components, as illustrated in Figure 1. We performed all tests on anesthetized Long-Evans rats. The rats' brain interacted with a dynamical system through a sensory interface and a motor interface. On the brain side, one microwire array delivered the microstimulation to the vibrissal representation of primary somatosensory cortex (S1) and a second microwire array recorded the neural signals from vibrissal motor cortex (M1). On the other side of the interface there was a simple and well-understood dynamical system: a simulated point mass moving over a horizontal plane within a viscous medium.

We began each experiment by collecting a “training” set of neural population responses to repeated presentations of different electrical stimulation patterns. We used these training data to implement a calibration procedure for establishing concurrently the encoding function of the sensory interface and the decoding function of the motor interface. Following the calibration procedure, we tested the competence of the interface (*test phase*) to drive the simulated point mass towards a goal location, which was defined by the central equilibrium point of a radial force field.

### Sensory-motor mapping

The purpose of the sensory-motor mapping is to set the parameters of the sensory and motor interfaces so as to approximate the desired force field. While force fields are continuous maps from position to force, the interface has a finite number of stimuli. Therefore, the mapping procedure must construct an approximation of the desired field with only a small number of vectors. To this end, we construct a cascade of three mappings: 1) a mapping from the position of the external device to one of selected stimuli; 2) a mapping from each stimulus to the evoked neural activity, and 3) a mapping from the evoked neural activity to the force acting on the external device. The first and last mappings are established by the interface software (i.e. sensory and motor interfaces), the second mapping is established by the properties of the neural structures that connect the stimulation and recording arrays.

In this first implementation, the sensory interface established a map from the position of the point mass to one of 4 stimulation electrodes (Figure 1A). The sensory mapping procedure (as detailed below) divided the workspace into 4 contiguous regions corresponding to a small “vocabulary” of 4 stimuli. At each iteration step, the interface algorithm selected the stimulus based on the region in which the point mass was located. The electrode delivered a train of 10 biphasic pulses (150 µA, 100 µs/phase) at 333 Hz [11], [12]. Larger vocabularies of stimuli can be generated by using a greater number of electrodes and by including electrode combinations. With a greater number of distinct stimuli, the workspace would be divided into smaller and denser regions, thus increasing the quality of the approximation of the desired continuous field. In a physiological system, the region of space that can activate a sensory neuron is called a “receptive field”. Here, the workspace of the sensory interface is divided into regions that are analogous to receptive fields: the mechanical system triggers an electrode when it passes by the region corresponding to that electrode.

The motor interface transformed recorded neural activities into force vectors applied to the simulated point mass (Figure 1B–D). A commercial spike-sorting algorithm (Rasputin, Plexon Inc.) decomposed the recorded neural signals into single-unit activities. We sorted 5–20 single units in each session from a 16 channel microwire array (average ± SEM across sessions was 13.69±0.48 units). The single trial responses of each neuron to the stimulation pattern consisted of a time series of spike counts computed in time bins of size Δt over a window of duration T·Δt, starting from the end of the stimulus. The neural population response was quantified as an array of such binned spike sequences. We found that post-stimulus windows of duration in the range between 100 and 600 ms binned at a resolution of Δt = 5 ms led to best performance of the interface (see below). Unless otherwise stated, in the following we present results obtained by running the interface using Δt = 5 ms and T·Δt = 600 ms. In this case, the input to the motor interface was a matrix with N rows and 120 (i.e. 600/5) columns (Figure 1B). During the test phase, the single-trial neural population response matrix was linearly mapped into the two components of a planar force vector.

### Dynamic shaping algorithm

In the following we describe the “dynamic shaping” algorithm for the concurrent calibration of the sensory and motor maps. The algorithm is defined by a set of 4 key parameters:

- N: number of recorded neurons as established by spike sorting
- T: number of time-intervals (bins) recorded on each neuron
- S: number of stimulation patterns (the stimulus vocabulary)
- R: number of repetitions of each stimulus pattern during the calibration procedure.

During the calibration each stimulation patterns was repeated R times and, accordingly, R×N neural responses were recorded. Each response was an array of T values: the number of spikes in each bin. The calibration responses were then represented as S×R N-dimensional vector functions:
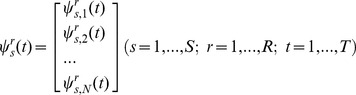
(1)From these calibration responses, we averaged the responses obtained from the repetitions of each stimulus, to extract S mean responses
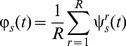
(2)Following the same notation, a neural response vector is an N-dimensional vector function
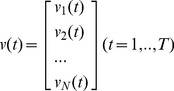
(3)The inner product of two neural responses is defined by extension over time bins and units of the Euclidean inner product:
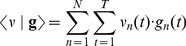
(4)The S mean calibration responses form a set of basis fields – a direct extension of the concept of basis vectors – that were used to approximate all recorded neural responses. In particular, each calibration response was approximated as a sum of mean responses:
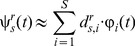
(5)To derive the combination coefficients 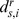, one takes the inner product of each side of Equation (5) with each basis function. This leads to S vector/matrix equations
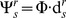
(6)where
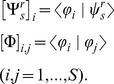
(7)Equation (7) can be solved for 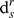 provided that 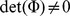 (if the projection matrix is singular, one can use a pseudo-inverse. But this does not seem to be a likely situation and was not encountered with any of our datasets).

With this, each calibration response was mapped respectively into an S-dimensional vector
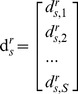
(8)Each response corresponds to a d-vector and vice-versa, each d-vector corresponds to a unique approximation of the response (the likelihood that two distinct signals map onto the same d-vector is vanishingly small). Therefore, we took the S-dimensional 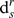 vectors as representations of the individual neural responses obtained after applying each stimulus.

### Motor interface

To calibrate the motor interface, we used principal component analysis (PCA) and extracted the two principal components that capture the greatest amount of variance in the set of the S×R calibration vectors, 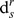. These two components are two S-dimensional arrays that form the rows of the 2×S projection matrix
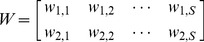
(9)This operator defines the two-dimensional plane with maximum variance over the set of S stimuli. The next step of the calibration procedure involved stretching the matrix so as to match the range of variation of the x and y components of the force vectors over the desired force field domain:
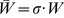
(10)The gain 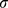 is a 2×2 diagonal matrix that scales the two-dimensional projections of the calibration recordings to cover the range of the desired force field, 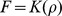. The field establishes a correspondence between the position, 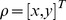, of the controlled object – in this first implementation a point mass – and a resulting force 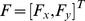. Here, we make the additional hypothesis that this field is invertible, which means that there is a function 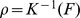 mapping force vectors to corresponding positions. This is obviously the case if the field is linear, as in 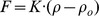 and the “stiffness” matrix is non-singular. The requirement of invertibility can be relaxed to a local and continuous form.

The two projection matrices, 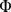 and 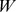, and the mean calibration responses, 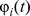, to all the stimuli generate a map from the data collected during the experiment to a corresponding two-dimensional force vector
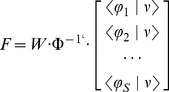
(11)This is a linear filter that operates in real time.

### Sensory interface

The sensory interface maps the instantaneous position of the controlled object onto one of the stimulation patterns in the calibration vocabulary.

This sensory interface performs a look-up operation:
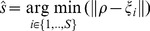
(12)that picks up the stimulus, 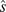, corresponding to the “calibration site” 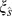 that is closest to the current position *ρ* of the controlled object. The calibration sites 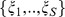
are the S locations:
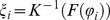
(13)where 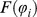 is the force derived by Equation (11) from the average response, 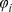, to the i-th stimulus in the vocabulary.

**Figure 2:**
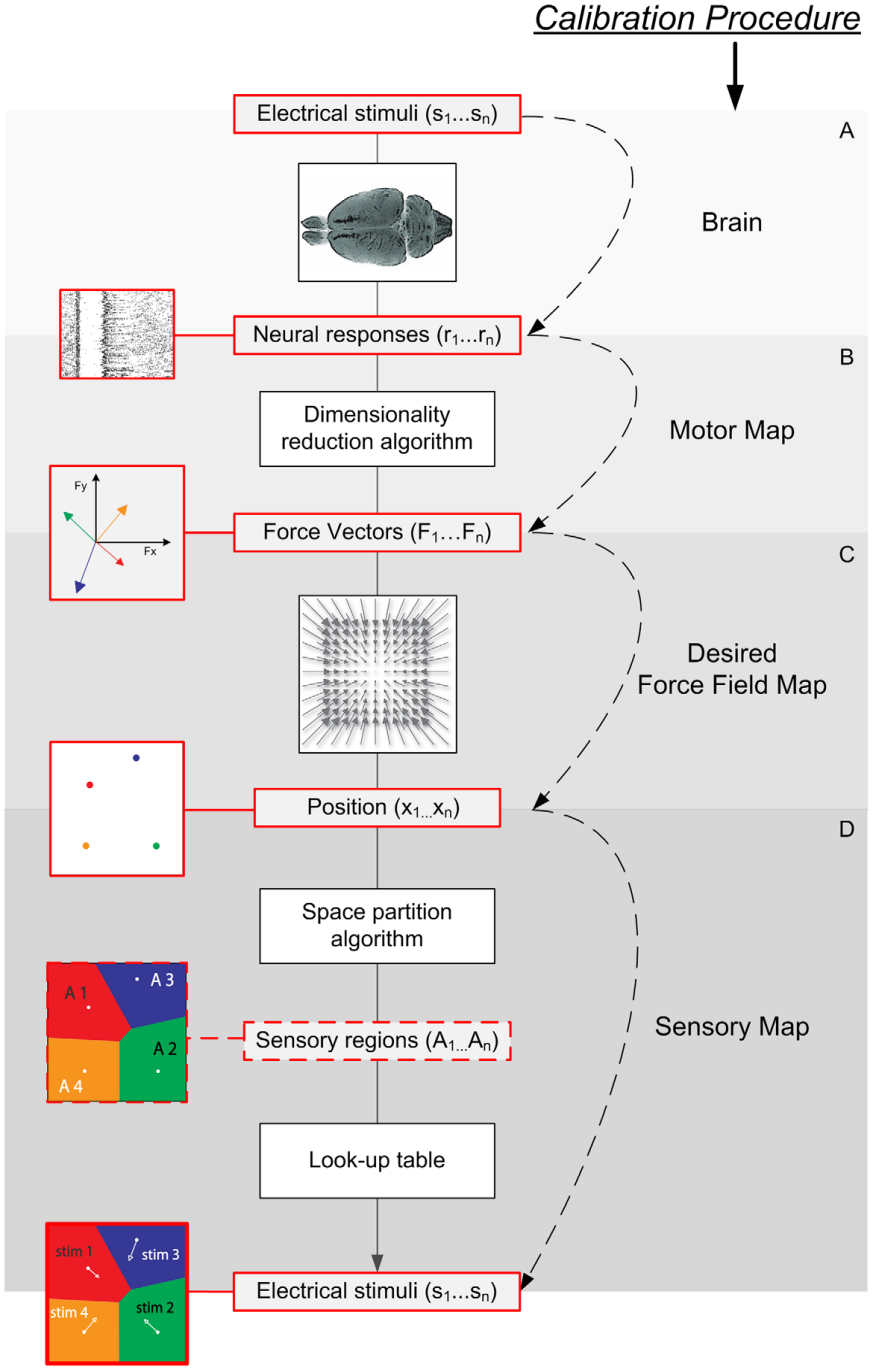
Calibration procedure scheme. The purpose of the calibration procedure is to set the parameters of the neural interface. The calibration consists of 4 steps. (A) *Recording a training set*. The calibration procedure is performed upon a *training set* (r_1_…r_n_) built by recording the neural activities evoked over multiple presentations (usually 100) of each of the *n* stimulation patterns (s_1_…s_n_ with n = 4 in this example). (B) *Motor Map*. The training set is also used to set the motor map. The spike trains from multiple neurons are reduced by PCA to two coordinates of a force vector. In this example the result of this operation is a set of 4 *template vectors*, each corresponding to a stimulation pattern. (C) *Desired force field map*. The chosen desired force field to be approximated (e.g. a continuous radial force field converging towards a central equilibrium point) establishes a relationship between the *n template vectors* and the *n* positions in the two-dimensional space. (D) *Sensory Map*. The *n* positions are used to partition the external device space by using a space partition algorithm (e.g. in this case a nearest neighbor map) and, as a consequence, *n* sensory regions (A_1_…A_n_) are defined. A look-up table connects each sensory region to a corresponding stimulation pattern. As a result, the sensory map converts each position of the space into a stimulation pattern.

In this first implementation, there were 4 distinct electrical stimuli, s_1_, s_2_, s_3_ and s_4_ and 4 mean corresponding neural responses, r_1_, r_2_ r_3_ and r_4_ (Figure 2A). Each mean neural response was a high-dimensional collection of spiking activities, which was reduced by the motor interface to the two coordinates of a force vector. Principal component analysis (PCA) performed this dimensionality reduction by extracting from each of the 4 mean neural responses recorded during the calibration phase the two principal components that capture the highest amount of signal variance. We scaled these two components so as to span the variance of the force vectors over the desired force field. This process resulted in a simple linear mapping, i.e. a gain matrix and an offset vector that, when applied to the neural response produced a force vector (Equation 11). In particular, the 4 mean responses collected during the calibration mapped to 4 template force vectors F_1_, F_2_, F_3_, and F_4_ (Figure 2B). The desired force field established a relationship between these template force vectors and 4 positions, x_1_, x_2_, x_3_ and x_4_ (Figure 2C). These 4 positions partitioned the space of the external device into 4 contiguous regions, A_1_, A_2_, A_3_ and A_4_, based on a nearest-neighbor map: a generic point x was associated to the region A_i_ if x_i_ was the nearest calibration position (Figure 2D). In this case, the sensory interface triggered the stimulus s_i_. It is straightforward to extend this procedure to an arbitrary number of stimuli for generating denser approximations of the desired force field.

### The dynamic neural interface generates an approximation of the desired force field

The concurrent operation of the sensory and the motor interfaces resulted in the realization of a force field that approximated a desired radial force field converging towards a central equilibrium point (Figure 2C). If one might assume that the recorded neural activity elicited by each stimulus remained invariant through time, then the field generated by the interface would be a piecewise constant approximation of the desired field. However, the inherent variability of neural activities observed after each repetition of an electrical stimulation pattern violated this assumption. This variability was mostly caused by background activities that interacted with the activities induced by the stimulus. In the anesthetized preparation, the background activities can be considered as random noise. In the alert animal, these activities may also contain a voluntary component. In this way the actual field is an additive superposition of the field approximation established by the interface with a random component induced by background neural noise. Extracting as much information about the stimulus as possible from the recorded signals is a key technical challenge for generating a controlled desired dynamical behavior with the bidirectional interface.

### The dynamic neural interface is able to drive a point mass to a target location

During the test phase, we probed the ability of the dNI to drive the simulated point mass towards a goal location, corresponding by design to the central equilibrium point of the *desired force field*. This is a simplified representation of a reaching movement, where the interface emulates the generation of a convergent force field similar to those observed after microstimulation of the spinal grey matter [4]–[6].

**Figure 3:**
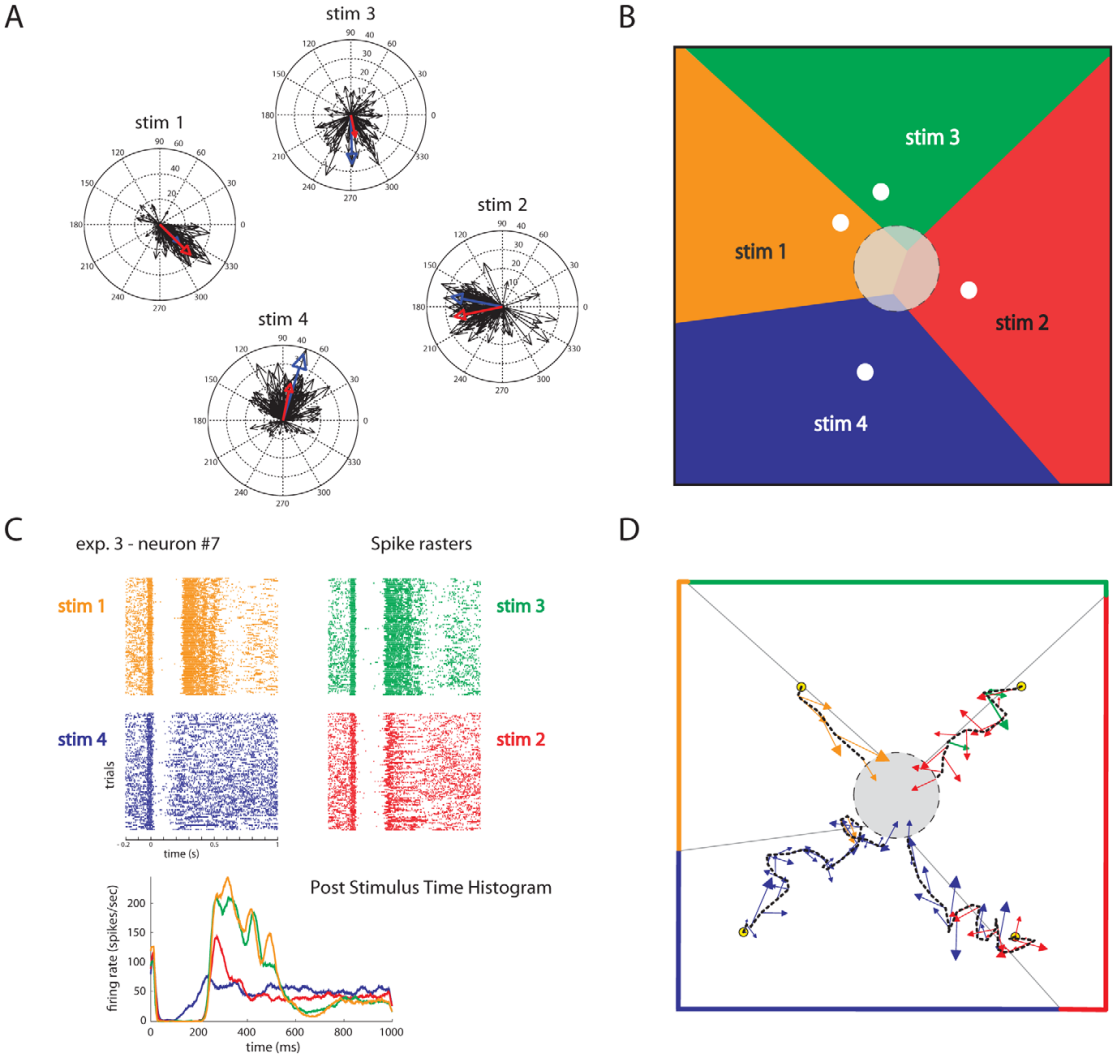
An example of motor and sensory interfaces and on-line closed-loop trajectories generated by evoked activity of a population of neurons. (A) The output of the motor interface during the test phase is represented as the force vectors (black arrows) generated during 100 “test set” repetitions of each stimulation pattern. The spread of the distribution of the single trial vectors represents neural variability at fixed stimulus. The small discrepancies between the angles of the template vectors computed during calibration (blue arrows) and the trial-averaged vector observed for each stimulus during the test phase (red arrows) originates from differences between training and test dataset due to neural variability and limited sampling. (B) A graphical representation of 4 sensory regions generated by the sensory interface. Each position of the point-mass is mapped onto a stimulation pattern and is color coded. (C) Spike rasters and Post Stimulus Time Histograms (PSTH) of a single neuron evoked by 4 different stimulation patterns, using the same color code as in panel B to distinguish responses to different stimuli. (D) Trajectories of the point-mass (dotted black line) generated on-line starting from 4 different initial points (yellow circles). For each trajectory, the force vector applied step by step by the motor interface to the simulated point-mass is indicated with a color code representing the stimulation pattern chosen by the sensory interface. In this example the forces were applied with an interval of 1 s and the point mass reached the target respectively in 6, 16, 25 and 29 s.

The dNI generated a movement of the simulated point mass (Figure 3D) by the following procedure:

1. The experimenter places the point mass at a starting initial state (position and velocity).
2. The sensory interface determines the stimulus to be delivered at that position, based on the nearest calibration site (see Equation 12). The stimulus is delivered.
3. The motor interface decodes the ensuing neural activity and derives the force vector to be applied to the point mass (see Equation 11).
4. The next position is computed by integrating the equation of motion of the point mass in a viscous medium (see Equation 15) for a short time (typically 1s).
5. The process is repeated from step 2 until the point mass reaches the region of equilibrium.

Because of the cortico-cortical pathways between stimulated and recorded populations [13], the neural population responses were clearly modulated by the stimuli (Figure 3C). However, the actual behavior of the interface contained a stochastic component due to the fact that each stimulation pattern, when repeated over different trials, caused a variable response in the recorded motor cortex. Part of the response variability in our anaesthetized preparation likely arose from trial to trial fluctuations in ongoing internal activity unrelated to the stimuli [14]. These trial to trial response variations resulted in a random time-varying component of the force field.

### Evaluation and optimization of the dynamic neural interface using information theoretic metrics and trajectory based metrics

The performance of the dNI likely depends upon information that the neurons make available for communication with the dynamical system, which in turn likely depends upon the temporal precision at which spike trains are considered [15], [16]. In particular, previous studies of neural encoding suggest that more information may be extracted from neural responses if they are examined with a relatively fine precision of the order of few to few tens of ms [17], [18] and that the optimal precision to extract information from neural activity may vary depending on the specific task or condition [19], [20].

In this study we therefore determined empirically the range of response parameters that maximized some measures of the quality by which the neurons can communicate with the rest of the system. The neural response r following the electrical stimulation was quantified as a time series of spike counts for each of the N neurons computed in T small time intervals of size Δt post-stimulation. The size of the bins Δt (corresponding to the temporal precision used to evaluate neural responses) and the parameters defining the time window duration (the number of time bins T and the offset of the post-stimulus window) are all arbitrary parameters that we attempted to set optimal according to some quantitative criterion. To study systematically how the performance of the dNI depends on the temporal parameters defining the neural response, we generated a set of “off-line” trajectories according to the following simulation procedure. At each step of the simulation, the position of the point mass was paired with the stimulation pattern associated with its nearest neighbor, as in the actual on-line experiment. Then, a recorded pattern was randomly drawn from an additional collection of neural responses to the 4 electrical stimulation patterns stored in the sensory interface.

Using the off-line trajectories, we estimated the amount of information that the neural population makes available to communicate with the dynamical system. This information was evaluated as the Mutual Information 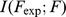 between the force vector expected to be generated by the electrical stimulation in a given trial (a template force vector corresponding to the mean force vector established during the calibration trials in response to the considered electrical stimulation, Figure 3A blue arrows) and the actual force vector obtained from the neural response in that trial.

**Figure 4:**
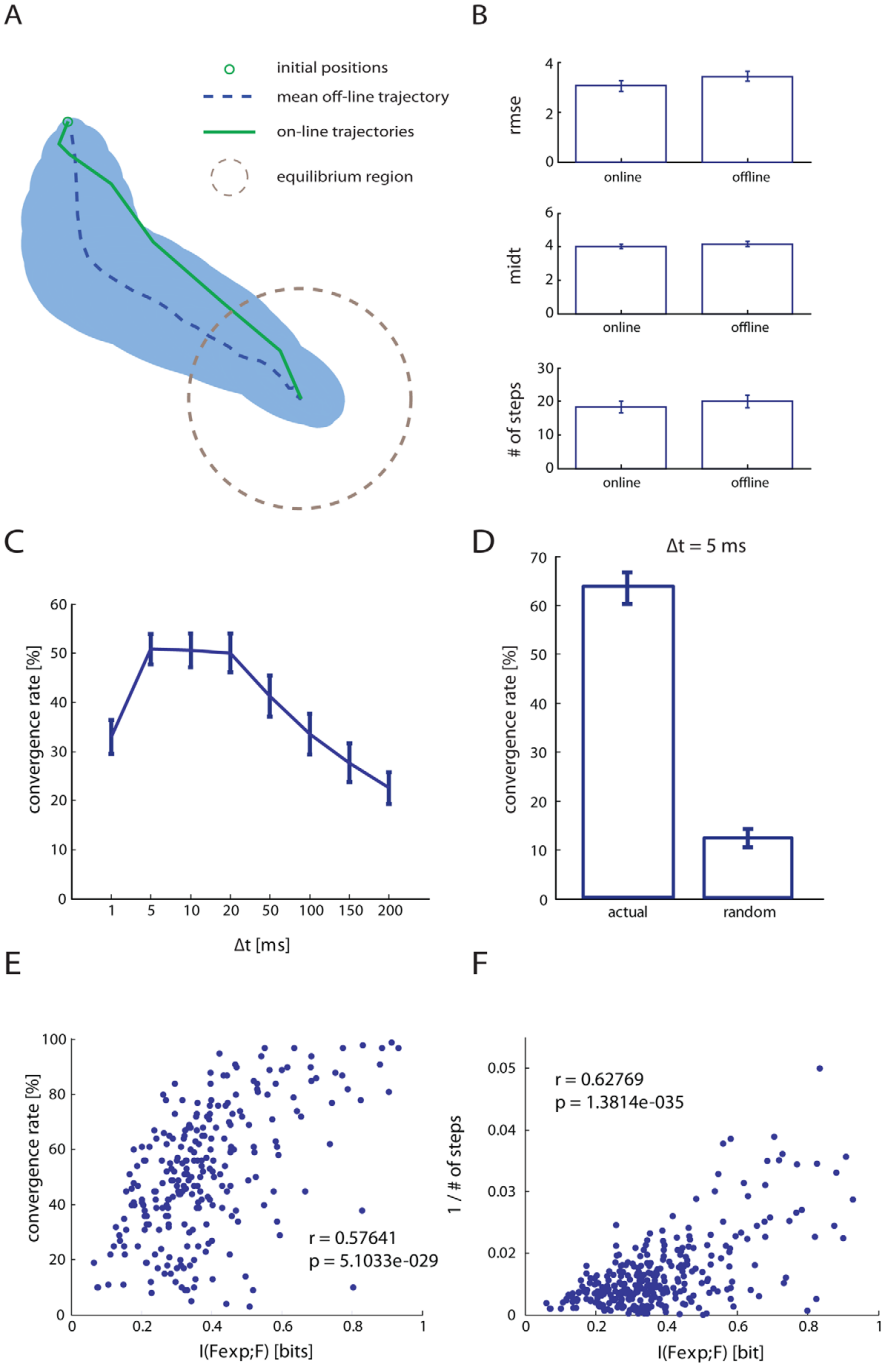
Off-line analysis of dependence of the dNI performances on the temporal parameters defining the neural response. (A) A closed-loop trajectory recorded on-line (green line) compared to 100 trajectories generated off-line (blue dotted line indicates the mean trajectory and shaded areas represent the p = 0.05 confidence region of trajectory). (B) We compared 70 converging on-line trajectories selected from 13 rats with 70 corresponding off-line trajectories using different parameters such as the root mean square error (RMSE) from the ideal trajectory, the mean integrated distance to target (MIDT) and the number of steps to convergence. Setting the time interval of 1 s between two consecutive steps, these values (mean±SEM: online = 18.2±1.6 and offline = 19.9±1.8) indicate also how long it took for this particular point mass to reach the target. Off-line and on-line behaviors were not significantly different (p>0.1; paired t-test), indicating that off-line simulated trajectories are representative of on-line behavior. (C) Mean convergence rate (CR) subtracted by the mean convergence rate obtained from a random choice of the stimulation patterns, calculated using different sizes for Δt. (D) Mean CR of the dNI calculated using Δt = 5 ms. The CR of the off-line trajectories is used to evaluate the performances of the interface, which is found to be maximal for small temporal resolutions (Δt≈5–10 ms). In particular, by using a bin size of 5 ms the mean CR is 6 times higher than the CR randomly built. The Mutual Information 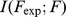 between the expected force vector and the actual force vector is highly correlated both to the CR (E) and to the inverse of the mean number of steps to convergence (F) calculated for all the simulated off-line trajectories.

**Figure 5:**
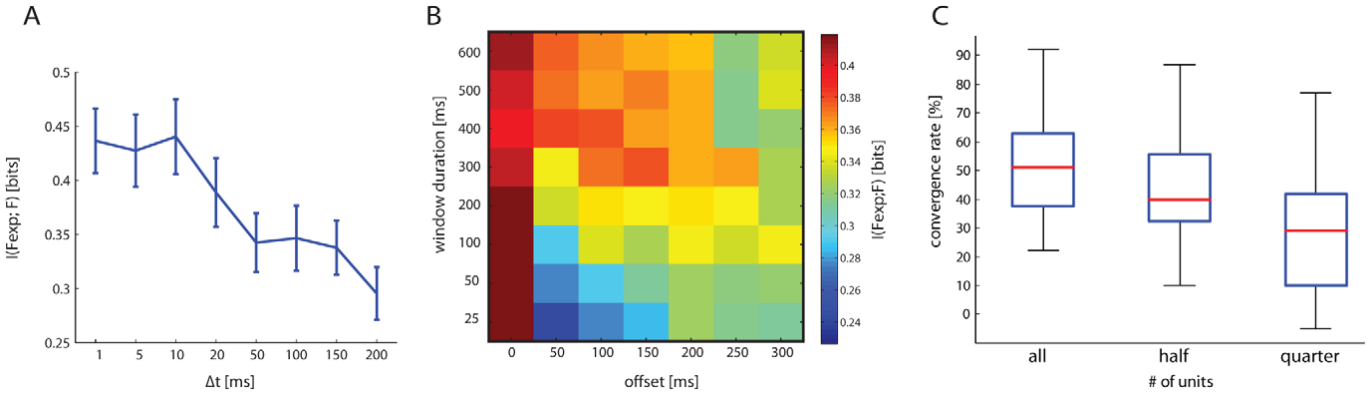
Role of recording parameters on Mutual Information measures of the performance of the dNI and dependence of performances on number of recorded single units. (A) Dependence of the Mutual Information 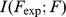 between the expected force vector and the actual force vector upon the temporal bin size Δt. Results were calculated using data computed in the response window with zero offset and 600 ms duration and are reported as average±SEM over all experimental sessions. Information was maximal for small bin sizes, such as Δt = 5–10 ms, meaning that the best performance of the dNI is obtained when recording neural activity with fine temporal precision. (B) Dependence of the Mutual Information 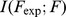, calculated with temporal resolution of Δt = 5 ms, upon duration (T·Δt) and offset defining the response window. Results are reported as average over all experimental sessions. (C) Convergence rate vs. population size. We compared the convergence rates when using all the neurons of each datasets with those using only half or one quarter of the units (subtracted by the mean convergence rate of trajectories randomly generated). Data are represented as box plots: red lines are the medians, lower and higher borders of the boxes indicate respectively the 25th and 75th percentiles, while the whiskers indicate the minimum and maximum value of each group. ANOVA test revealed that only the quarter case is statistically different from the other two (p = 3.3552e-006).

We found that the really critical response parameter was the temporal precision Δt at which spikes are sampled (Figures 4C and 4D). A fine temporal precision Δt≈5–10 ms was needed to obtain high Information values. Using coarser temporal precisions of 50 or 100 ms led to dramatic decreases of the Information values (Figure 5A). Figure 5B reports the results of how the Information 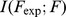, averaged over all sessions and calculated using a sampling precision Δt = 5 ms, depended upon the windows duration T·Δt and upon the offset value defining the response window. Information was very stable in the range T·Δt≈25–600 ms. The fact that the interface performs well also for decoding windows as short as few tens of ms encourages us to believe that it will be possible to push the dNI technology towards implementing feedback which is rapid enough to control real life motor tasks.

Moreover, there was a highly significant correlation (p<10e-9) between the Information 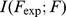 and both the convergence rate (the percentage of trajectories that converge into the target) and the inverse of the mean number of steps to convergence of the off-line dNI trajectories (Figure 4E–F). As a result, the performance of the dNI was maximal for fine temporal precisions: the convergence rate peaked for Δt≈5–10 ms (Figure 4C). At Δt = 5 ms, the convergence rate of the dNI was on average 6 times higher than the convergence rate obtained with a purely random choice of the electrical stimulus to be applied (Figure 4D), demonstrating that the neural information had a sizeable impact on the dNI dynamics. These results suggest that precise spike timing is not only crucial for communication within the nervous system [16], but it is also important for bidirectional communication between external effectors and the nervous systems.

The impact of the Mutual Information provided by the neurons participating in the dNI upon the performance of the dNI was further investigated by studying the relationship between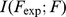 and the convergence speed of the dNI on the off-line simulated trajectories. For each set of possible response parameter and experimental session, we computed the mean number of steps needed for the trajectory to converge and the probability of reaching convergence to the center of the force field (averaged over 100 off-line-generated trajectories) with these response parameters and we correlated it with the Information computed in the same conditions. In sum, the empirical finding was that higher Information values corresponded to faster and more reliable convergence of the dynamical behavior and all measures pointed to the same range of neural response parameters being optimally efficient for dNI operation.

We also evaluated how the performance of the interface depended upon the population size by comparing the convergence rates when using all the neurons of each datasets with those using only half or one quarter of the units. The average number of recorded neurons during each experimental session was 13.69±0.48 (mean±SEM over all sessions). For each dataset, we randomly selected (out of *nA* recorded units) *nH* and *nQ* units for the calculation of the performance with half and one quarter units, with *nH* and *nQ* being the approximation to the closest integer of *nA/2* and *nA/4*, respectively. For each selection of the subpopulation, we subtracted the obtained convergence rate by that obtained from a random choice of the stimulation patterns (as we did when analyzing the performance of the entire population). Figure 5C shows that a decrease in performance is observed only when reducing the population size to one quarter of the recorded one. Convergence rates with one quarter neurons are statistically different from the rates in the other two cases (p = 3.3552e-006, ANOVA), while the performances with all and half neurons were not statistically different (p>0.1, ANOVA). This suggests that using multi electrode recording arrays is useful for the performance of the system.

Finally we used different performance metrics to compare on-line trajectories with off-line simulated trajectories to evaluate if the off-line dataset could be used to simulate and study in more detail on-line behavior. To perform this comparison we selected 70 converging on-line trajectories selected from 13 rats and 70 corresponding off-line trajectories. In particular we calculated the root mean square error (RMSE), the mean integrated distance to target (MIDT) and the number of steps to convergence. For the calculation of RMSE, we first computed for each trial i the ideal trajectory 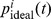 as the one sharing the initial point with the actual trajectory, but evolving with a force 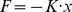. Then, for each trial i we computed the root mean square error as 
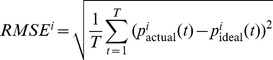
 with T being the maximum duration of the trial and 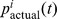 the actual position of the point-mass at time t. We computed MIDT as the average distance from the target. For each trial i, being 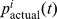 the position of the point mass at time t and 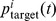 the position of the target we define: 
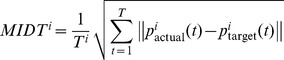
. Because the target corresponds to the origin of the plane, MIDT is simply the length of the trajectory normalized by its converging time.

As reported in Figure 4B, we found no significant differences in the computation of RMSE, MIDT or number of steps to convergence between on-line and off-line data (t-test, with p = 0.17 for RMSE, p = 0.41 for MIDT, p = 0.5 for number of steps). The consistency between the off-line open-loop simulated trajectories and the actual closed-loop trajectories recorded on-line during the experiment suggests that the parameters set optimally by generating offline simulated trajectories from calibration data will be optimal also for running the same interface online. In this respect Mutual Information is an advantageous optimization metrics during calibration, because the corresponding evaluation of the inverse number of steps requires running a larger number of simulated trajectories and would thus be computationally slower.

## Discussion

With few notable exceptions [21]–[25], the development of neural interfaces has proceeded along two separate tracks. There are sensory interfaces, such as the cochlear implants [26] that transform external physical events into neural stimuli for the brain and there are motor interfaces that decode activities from cortical regions to generate commands for external devices [1], [27]–[29]. However, the efficiency of biological motor behavior rests upon the seamless integration of sensory information and motor commands. This integration occurs both in our deliberate and conscious responses to external stimuli and in hardwired reflex responses organized by the neural circuitry of the spinal cord. In fact, the voluntary motor commands originating from the highest brain centers operate upon the world by modulating the activities and the response properties of the spinal networks. Here, we have taken a first step towards the design of a brain-machine interface that emulates the same basic principle: the interface has a sensory and a motor component whose direct interaction generates a system of automatic responses, which are to be modulated by volitional activities. In this sense, our proposed architecture draws inspiration from the natural “neural interface” that all vertebrates are endowed with: the spinal cord.

Unlike its biological counterpart however, the proposed interface is not connected to a musculoskeletal system, but can act over a broader family of dynamical systems. In this example, we chose a simulated point-mass moving within a viscous fluid. The interface generates position-dependent forces converging to a stable equilibrium point. This simple framework highlights an important issue in the design of brain-machine interface: the boundary between neural and artificial control. The parameters of the external system – in this case the viscous and inertial matrices – may result from a combination of passive physical elements and feedback control components. There is therefore an important role of the engineering design in establishing the dynamical properties of the external device, as it is seen by the neural system through the interface.

### Force fields in motor behavior

The concept that force fields afford a representation of the motor output in the spinal cord was first expressed in the aforementioned stimulation studies [4]–[8]. However, the mechanistic concept behind this representation can equally well characterize a variety of other observations, including some of the most classical ones. The stretch reflex first described by Sherrington [30] is one the clearest examples. Another example is spinal pattern generators that produce a different type of field, a field inducing a cyclical motion of the limbs. Grillner and coworkers [9] offered a compelling model of locomotion pattern in the lamprey, and in both cases the rhythmic activity is sustained by a phase-shift between the state of motion and the consequent forces. While the experimental tests in the current paper have been focused on the enforcement of equilibrium-seeking behavior, different behaviors are programmable through the approximation of different force-fields.

The description of the bidirectional neural interface as a force-field has a conceptual rationale in the causality of mechanical interactions between a control system and its environment [31], [32]. At the interface with the environment, a control system may act either as a generalized admittance, determining a state of motion in response to an applied force, or as a generalized impedance, determining a force in response to an applied state. Considerations about neuromuscular mechanics suggest the second case as more appropriate, because the mapping from state (position and velocity) to force is typically well defined but not invertible. In this sense too, the architecture of the interface reflects the organization of the biological motor system. However the extent of the similarity may vary depending on the structure that is being controlled. The dynamical parameters – for example the mass and viscosity – may be characteristics of the physical system that is been controlled by the interface. But they also may be – at least partially – introduced in the interface algorithms to shape a desired behavior. For example a virtual mass and a virtual viscosity can be added in parallel to the physical system to increase stability and modify the resulting trajectories.

Intelligent and purposeful motor behavior involves the ability to react to unexpected perturbations and to change planning goals. In this respect, the study presented in this report represents a preliminary step towards the development of an interface that facilitates exploration and adaptation providing its users with the possibility to modulate a field of forces. Even if the concept of controlling a limb by shifting its equilibrium position is not new [33]–[35], in the context of BMIs this is a radically new platform compared to current approaches based on decoding – instant by instant – the desired state of motion of the connected device, such as, for example, a robotic arm. Consider a reaching movement with a prosthetic hand. As the hand moves towards the target an obstacle is encountered that triggers a correction. The standard decoding method requires recreating an entire path that circumvents the obstacle and reaches the final target. In contrast, a field-based approach, reprogramming the path may be limited to shifting the hand position to a point that is clear of the obstacle and then let the field guide the hand towards the target without further reprogramming.

### Somatosensory perception

While early BMI studies were mostly focused on decoding motor cortical activities [29], [36], more recently there has been a growing interest for evoking somatosensory perception by electrical stimulation. For example Weber and co-workers are pursuing the stimulation of dorsal root ganglia, recreating patterns of evoked responses in somatosensory-area [37]. Recently, Venkatraman and Carmena [38] were able to stimulate neurons in the rat barrel cortex and to produce the sensation of an object being swiped by the whiskers. More recently yet, Nicolelis and coworkers were able to integrate in BMI motor cortical decoding with artificial tactile sensing elicited by microstimulation of S1 [21]. These results are consistent with earlier observations by Romo and coworkers who demonstrated the possibility to induce tactile sensation analogous to finger touch in monkeys [39]. Based on the available evidences, we expect the electrical stimuli generated by our interface to be adequate to induce somatosensory perception in the alert animal. Since we are stimulating in the barrel cortex, we predict – after Venkatraman and Carmena [38] – that the stimuli would induce perceptions analogous to whisking an object. However, in a brain-machine interface the ultimate goal would be to produce sensations corresponding to the state of an artificial device, such as a food feeder, whose structure may or may not resemble that of a biological limb. Understanding how the somatosensory system may adapt the perceptual correlate of electrical stimuli is a future research goal, beyond the scope and reach of the present study. Here, we focused on the production of automatic responses in the form of preprogrammed force fields, in the perspective that these responses may be both accessible and modifiable by volitional commands. Studies of current interfaces provide ample evidence demonstrating the ability of the mammalian brain to modulate the activities of populations of cortical neurons in different brain areas [27]–[29], [40], [41]. To the extent that this circuitry can be accessed and purposefully modulated by voluntary neural commands, the dNI will offer its user with the possibility to achieve motor goals in a stable manner and without the need for constant on-line supervision. At this time, however, the possibility that the force field produced by the interface may be accessible to volitional control remains to be demonstrated by additional experiments with alert animals. In particular, it will be critical establishing what field parameters may be modified by volitional inputs converging upon the neural structures that determine the output of the interface. We need to stress that the particular case of a convergent field is not the only that can be implemented and that has functional relevance. For example, a force field can be programmed to have rotational structure so as to induce cyclical motions of the controlled objects. Parallel pattern of forces, on the other hand, may approximate the control of a contact force. The simple case of the viscoelastic force field in our task provides the mathematical basis for generating stable trajectories – i.e. trajectories that converge to a nominal path in exponential time if displaced by an unexpected perturbation. In addition to expanding the behavioral repertoire of NIs, the bidirectional interface establishes a new venue for investigating the mechanisms of neural plasticity through a controlled exchange between cortical structures and a virtually unlimited repertoire of dynamical systems implemented either in hardware or by computer simulation.

## Methods

### Ethics statement

This study was carried out in strict accordance with the Italian law regarding the care and use of experimental animals (DL116/92) and approved by the institutional review board of the University of Ferrara and by the Italian Ministry of Health (73/2008-B). For all experimental procedures, rats were anaesthetized with a mixture of Zoletil (30 mg/kg) and Xylazine (5 mg/kg) delivered intraperitoneally and all efforts were made to minimize suffering.

### Neurophysiological procedures and experimental set-up

The experiments were carried out on 13 male Long-Evans rats, weighting 350–400 g and for the entire duration of the experiment, anesthesia was maintained with supplementary doses of anesthetic (intra-peritoneal or intra-muscular) such that a long-latency, sluggish hind limb withdrawal was sometimes achieved only with severe pinching of the hind foot. The anesthetized animal was placed in a stereotaxic apparatus (Myneurolab). A craniotomy was made, using a micro drill, over the primary somatosensory cortex (S1) and primary motor cortex (M1) whisker representations of the same hemisphere. To place the stimulation array, a small craniotomy (2×2 mm) was made in the parietal bone to expose the barrel cortex, which was identified according to vascular landmarks and stereotaxic coordinates [42]–[44]. The dura mater was not removed because the electrodes were sufficiently rigid to pass through it. The placement of the electrodes was tested and confirmed by recording the neuronal responses to manual whisker stimulation. The arrays were lowered perpendicular through the cortical surface using a hydraulic microdrive (2650, Kopf) at depth between 500 and 900 µm from the pia (granular layer) [45]–[47].

**Figure 6:**
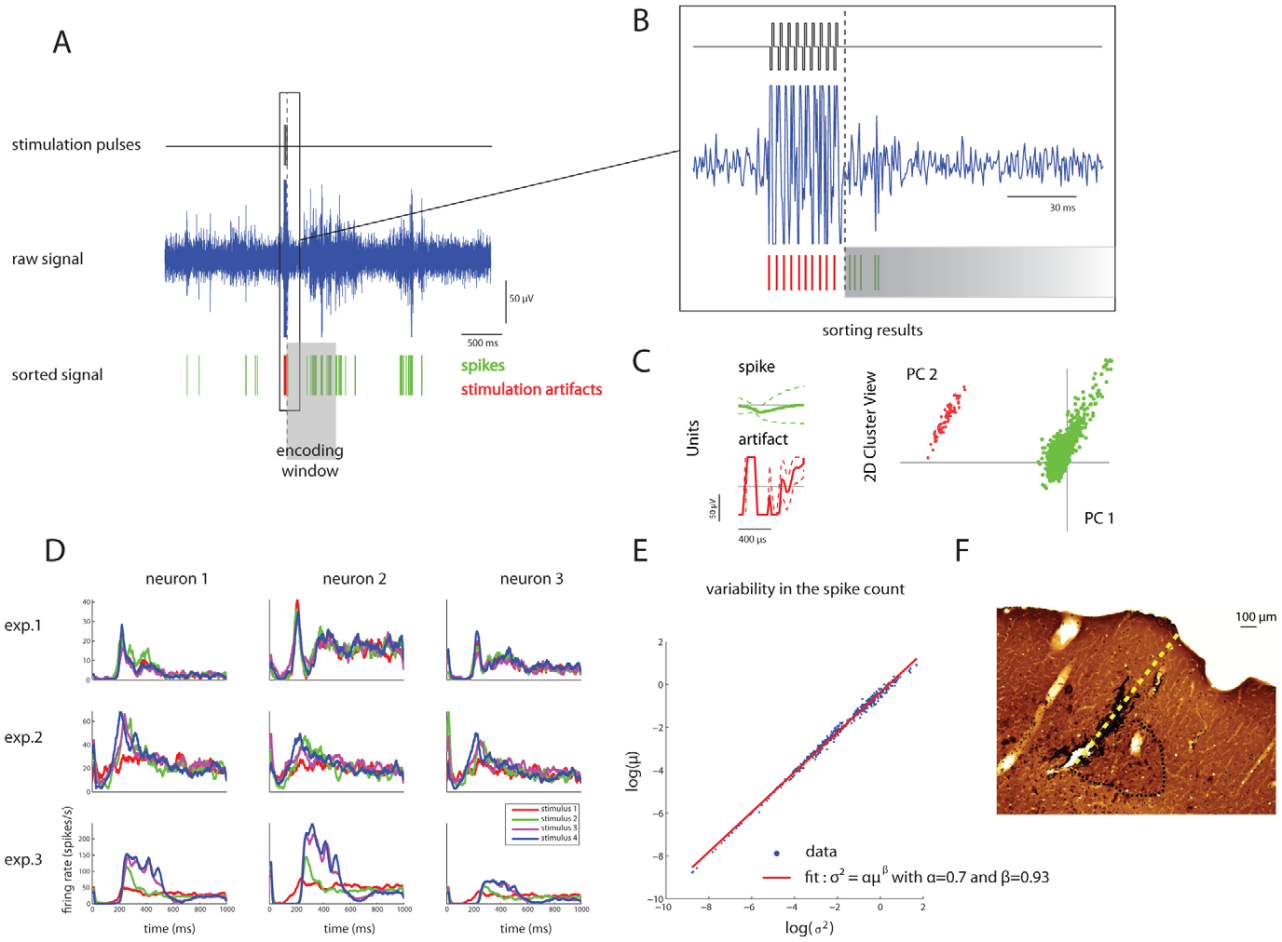
Recorded neural activities in M1 evoked by electrical stimulation in S1. At the beginning of each experimental session a series of electrical stimulation patterns is delivered and a sorting procedure is performed on the raw neural signal to identify both the stimulation artifacts and the single unit activities. Panels (A) and (B) show a portion of a raw signal close to a stimulation event. The sorting procedure is able of identifying the stimulus artifacts (red lines) and the spike occurrences (green lines). Panel (C) shows the unit templates used by the sorting algorithm (left) and a representation of the sorted data onto the first two principal components plane (right). (D) Post Stimulus Time Histograms (PSTH) of neural evoked responses of a subset of three neurons selected from three experiments. The color code represents different stimulation patterns. (E) Scatter plot of variance vs. mean of spike counts (computed in sliding 20 ms long post-stimulus windows) of all pooled data points across units and sessions. This measure is a relatively standard measure of cortical response variability. The best-fit power law curve (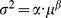 with α = 0.7 and β = 0.93) is plotted with the best fit parameters. These data are at the most reliable end of the range of response variability reported in the cortical literature. (F) CO stained section (AP = −3.3 mm from bregma) of the rat brain with microelectrode track. The black dotted line indicates the boundary of the barrel. The perpendicular length from the tip of the electrodes (the center of the hole) to the cortical surface measured 730 µm.

To insert the recording array, the frontal cortex was uncovered at 0.5 mm rostral and 0.5 mm lateral to bregma, and the vibrissal representation was exposed, at coordinates consistent with previous maps of the M1 whisker representations [12], [43], [48]–[50]. In preliminary experiments, we conducted intracortical microstimulation (monophasic cathodal pulses, 30 ms train duration at 300 Hz, 200 µs pulse duration with a minimum interval of 2.5 s) to evoke whisker twitches, at high threshold intensities, between 1.5–1.8 mm below the cortical surface. This depth was found to correspond to the layer V of granular cortex. The microwire array was lowered perpendicularly into the cortex to layer V at sites ranging from 1.0 to 2.5 mm lateral and 1.0 to 3.0 mm rostral to bregma. Also in this case the dura was not removed and was kept moist with a 0.9% saline solution. The effectiveness of the placement of stimulation and recording arrays was verified by computing peri-stimulus time histograms of neural responses to the different stimulation patterns (see Figure 6A–D for an example).

For both recording and stimulation procedure we used 16 polyimide-insulated tungsten electrodes microwire arrays (50 µm wire diameter, Tucker-Davis Technologies), configured in two rows of 8 electrodes each (250 µm electrode spacing and 375 µm rows separation) and placed over the primary somatosensory cortex (S1) and primary motor cortex (M1) whisker representations of the same hemisphere. Placement of electrodes was later confirmed by histological section.

The intracortical microstimulation (ICMS) consisted of trains of 10 biphasic pulses, each phase lasting 100 µs, delivered at 333 Hz with amplitude of 150 µA. Each stimulation train was delivered throughout two adjacent electrodes of the stimulation array using a programmable 8 channel stimulus generator (Stg4008, Multichannel Systems) built with a stimulus isolation unit for each output channel. Software-generated TTL triggers were used both to start the stimulation pattern and to store the stimulus timing in the recorded neural signals.

The recording microwire array was lowered perpendicularly into the cortex using a hydraulic microdrive (2650, Kopf) and extracellular neuronal discharges were recorded using a multichannel recording system (Map system, Plexon Inc.) with a sampling frequency of 40 KHz per channel.

During the experimental sessions an on-line PCA-based sorting procedure (illustrated in Figure 6C) was performed using commercially available software (Rasputin, Plexon Inc.). Time stamps of identified units were sent in real-time via local LAN to custom-made software developed in Matlab (Mathworks®) to translate the input neural signal into output stimulation triggers according to the behavior of the simulated controlled system.

We ensured that the neural responses used to guide the interface did not contain a component which reflected an electrical stimulation artifact rather than true neural response by the following steps: (i) we used only responses collected after the stimulation artifact had ended (i.e. the onset of neural response activity in each calibration trial and test trial started after the stimulation artifact ended) (ii) the templates of the on-line spike sorting procedure were established without including data collected during electrical stimulation (iii) we further verified by visual inspection that spikes identified near the onset had the same amplitude and shape of that identified far from the electrical stimulation (Figure 6A–C).

### Histology

At the end of electrophysiological session, DC of 5 µA for 10 s was passed through electrodes placed both at the beginning and at the end of the array, to mark its position. The current produced a lesion that was easily seen in cytochrome oxidase-stained histological sections. When the acute experimental phase was completed, the animals were deeply anesthetized with Isoflurane and transcardially perfused with 500 ml of 0.1 M-phosphate buffered saline (PBS) with 0.9% NaCl at 37°C followed by a 1l cold buffered solution of 2.0% paraformaldehyde, 1.25% glutaraldehyde and 2.0% sucrose (pH 7.4). The brains were removed from their skulls, coronally transected at the level of bregma and then postfixed overnight at 4°C. The caudal portion, including S1, was saturated in 20% sucrose, then 30% sucrose until it sank. Coronal sections of frozen brain (60 µm thick) were cut on a sliding microtome (SM2000R, Leica) to determine the depth of microelectrodes tip. The sections were processed for cytochrome oxidase (CO) according to previous reports [51], [52] to identify layer IV. Sections were washed three times in a 0.1 M PB solution and then incubated at 37°C in a cytochrome-C oxidase staining solution containing 4% sucrose, 0.05% DAB, and 0.05% cytochrome C (Sigma Laboratories), until barrels were clearly delineated. Then sections were washed in PBS and mounted on slides. Mounted sections were dehydrated in a series of alcohols, defatted in xylene and coverslipped.

CO stained sections were observed under brightfield illumination with Olympus BX51 microscope (Olympus) interfaced with a color video camera (CX-9000) and with a NeuroLucida system (MicroBrightField) (Figure 6F). Using a 10× objective, live color images of the histological material were displayed on a high-resolution video monitor. The boundaries of the barrels were drawn using the image on the screen and the depth of the electrolytic lesions was measured by the Neurolucida software.

### Simulations of the dynamic system interacting with neural activity

In this implementation, the device interacting bidirectionally with neural activity is a simulated point mass in a viscous medium. Typical values for the mass (M) and viscosity (B) were 10 Kg and 15 N•s/m. A linear force field 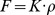 results in the linear differential equation
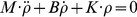
(14)with an isotropic stiffness (K) of 4 N/m, the ideal system driven by the noiseless linear field was slightly over-damped (damping ratio 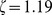). While the choice of these parameters is arbitrary, in a practical implementation, the parameters of the viscoelastic field (here, *K* and *B*) should be selected based on the desired time constant of the payload's motion. As the interface implements a piecewise constant approximation of the linear field, 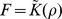, corrupted by random background activity, the stability properties afforded by the desired continuous field can only be considered as an optimal limit. This first realization of the interface has some notable limitations. One is that the control law generates an output force in response to a position input. In a more complete system, the input should convey not only position, but state information, that is position and velocity. Here, the derivative component of the controller is a fixed property, expressed by the term 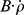 in the dynamics equation. Another obvious simplification is in the choice of a point mass (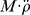) for controlled object. A mechanical arm is generally characterized by a non-linear differential equation. However, the second order linear ordinary differential equation (Equation 14) is used in robotics to represent the error dynamics of non-linear systems controlled by proportional-derivative (PD) methods [53]:
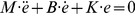
(15)with 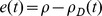 (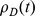 is a desired trajectory). In our framework, this PD control law can be reformulated as
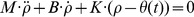
(16)where 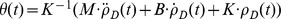 is a time varying function to be supplied by the voluntary input to the interface. In this case, the dNI would provide stability to a desired movement in a way analogous to the combined influence on limb movements of muscle mechanics and feedback mechanisms of the spinal cord. Therefore, while the form of Equation 14 is quite simple, it also expresses a fundamental mathematical representation for control.

By tuning the sensory and motor interfaces to approximate a predetermined force field, the dNI establishes an automatic behavior. The neural connections between the stimulated and the recorded populations determine the force to be generated at each position in the field. However, the recorded activities are also affected by inputs from other brain areas. In the alert brain, these additional inputs provide a pathway for the volitional commands to modulate the dynamics of the interface. To see this, suppose that the output of the interface is the programmed force field, 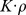 (where *ρ* indicates the radial distance from the origin of the plane upon which the point mass moves) plus a force component, 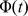 generated by a volitional command. The net force is then
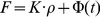
(17)This can be re-written as
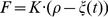
(18)where
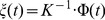
(19)is a time-varying equilibrium point. Thus, the dNI architecture provides a way to integrate voluntary commands with preprogrammed automatic responses so as to generate dynamically stable movements. A computer simulation study of the relationship between Information in neural activity, the mechanical parameters of the dynamical system and the performance of the neural interface is reported in [54].

### Calculation of Mutual Information between expected and actual force vectors

As explained in Results, we considered the Mutual Information that the recorded neurons provide to guide the dynamic system. The latter was evaluated as the Mutual Information 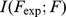 between the force vector expected to be generated by the electrical stimulation in a given trial (corresponding to the template force vector established during the calibration trials in response to the considered electrical stimulation) and the actual force vector 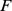 obtained from the neural response using the algorithm described in the above Section:
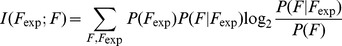
(20)where 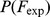 is the probability of presenting an electrical stimulation that leads to an expected force 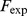, 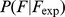 is the probability of obtaining in a given trial a force vector 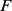 when presenting an electrical stimulation that leads to an expected force 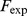, and 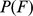 is the probability of obtaining in a given trial a force vector 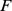 unconditional to the type of electrical stimulation applied. High (respectively low) values of 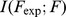 indicate instead a near-deterministic (respectively near-random) relationship between the force provided by the neurons and the one needed for guiding the dynamic system.


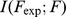
 was computed from the data as follows. Since there is a one-to-one correspondence between 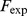 and the type of electrical stimulation pattern, and since Mutual Information is invariant to monotonic transformations or relabeling of the variables, the patterns 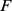 were labeled with the same index s (s = 1, …S) that indexes the electrical stimulation patterns. Then, the conditional probabilities of 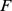 to each stimulation pattern s were computed as frequency-of-occurrence histograms from the trials to stimulus s. The values of the components 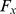 and 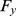 of the force 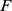 were discretized into five equipopulated bins in order to facilitate the sampling of the empirical probability histograms. Then, the probability histograms were plugged into the above equation for 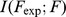 and its value was computed numerically. It is well known that, because the empirical probabilities are estimated from a limited number of trials, these empirically obtained Information measures still suffer from an upward systematic error (bias) due to limited sampling [55]. We corrected for this bias as follows. First, we used a simple analytical procedure [56] to estimate and subtract out the bias of each Information quantity. We then applied the “shuffling procedure” described in [55]–[57], which greatly reduces the bias of multidimensional Information estimates. We then checked for residual bias by a “bootstrap procedure”: stimuli and responses were paired at random, and the Information for these random pairings was computed. Because in this random case the Information should be zero, the resulting value is an indication of a residual error. In this study we found (data not shown) that the bootstrap estimate of this residual error was very small and much smaller than the Information values obtained for optimal neural response parameters, indicating that our estimates of 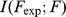 were reliable.

## Supporting Information

Video S1 The video clip describes the calibration procedure and the operation of the dynamic neural interface.
(WMV)
